# Evaluation of pulmonary B lines by different intensive care physicians using bedside ultrasonography: a reliability study

**DOI:** 10.5935/0103-507X.20190058

**Published:** 2019

**Authors:** Juliana Rodrigues Vieira, Marcela Rangel de Castro, Thaís de Paula Guimarães, Aldo José Tavarez Pinheiro, Ana Clara Tiso Costa Figueiredo, Bruna Jacomini Martins, Daniel Reis do Carmo, Wesley Academes Oliveira

**Affiliations:** 1 Hospital Felício Rocho - Belo Horizonte (MG), Brasil.; 2 Faculdade da Saúde e Ecologia Humana - Vespasiano (MG), Brasil.

**Keywords:** Lung/diagnostic imaging, Ultrasonography, Reproducibility of results, Respiratory insuficience/diagnostic imaging, Critical care

## Abstract

**Objective:**

To evaluate the agreement between intensive care physicians with similar training in the use of bedside lung ultrasonography in identifying pulmonary B lines, visualized in real time, to verify the reproducibility of the method.

**Methods:**

A total of 67 patients with some ventilatory deterioration identified within 12 hours after a pulmonary ultrasonography in the period from November 2016 to March 2017 were analyzed, and all were admitted to an intensive care unit of a private hospital in Belo Horizonte, Minas Gerais. The lung ultrasonographies were performed by three different professionals, termed A, B and C, and the time interval between each lung ultrasonography was less than 3 hours. The only visualized chest zones were the anterior and lateral, defined as right and left anterior (1) zones (Z1R and Z1L, respectively), which were delimited by the clavicle, the sternum and the horizontal line perpendicular to the xiphoid process and anterior axillary line. The right and left lateral (2) zones (Z2R and Z2L, respectively) covered the lateral area between the anterior and posterior axillary lines, with the lower limit being the same horizontal line corresponding to the height of the xiphoid process. A lung zone was considered positive for B lines upon visualization of three or more of these lines, suggesting the presence of alveolar-interstitial syndrome. Using the Kappa value, we evaluated the agreement among the four zones according to the execution of each pair of professionals (AB, AC and BC).

**Results:**

Approximately 80% of the areas that were visualized showed a moderate to substantial agreement, with the Kappa values ranging from 0.41 - 079 (p < 0.05; 95% CI). The highest levels of agreement occurred in the upper zones Z1R and Z1L between subgroups AC and BC, with a Kappa of approximately 0.65 (p < 0.001). In turn, Z2L showed one of the lowest agreements, with a Kappa of 0.36.

**Conclusion:**

The possible limitation of an examiner-dependent effect on lung ultrasounds was not found in this study, suggesting the good reproducibility of this diagnostic modality at the bedside.

## INTRODUCTION

Lung ultrasonography (LU) is a noninvasive imaging technique that has increased in popularity, especially among intensive care and emergency physicians. It can provide relevant and accurate complementary information in the diagnostic evaluation of critical patients with acute respiratory failure.^1,2^ Its availability has increased, making this method one of the first diagnostic modalities that can be applied to bed-bound patients,^2^ and its use also requires accessible professional training.

The exam has a sensitivity greater than 90% in the assessment of alveolar-interstitial syndrome (AIS) and is also useful for the elucidation of other entities, such as pleural effusion and consolidation.^3,4^ All of these diseases reduce pulmonary aeration, which generates visible and characteristic patterns on an LU. In addition to the potential to diagnose or exclude pathologies, the execution of LU is quick and avoids risky transport of patients, enabling early monitoring and interventions.^5^

The aerated pulmonary parenchyma is represented on the LU by hyperechoic and static horizontal lines, which are repeated at regular intervals and are called A lines.^6^ The presence of interstitial thickening, such as pulmonary edema and fibrosis, generates vertical and hyperechoic lines on the LU that move in synchrony with the respiratory cycle and are called B lines.^2-7^ Some studies, such as those of Volpicelli et al. and Lichtenstein et al., suggest the presence of AIS when three or more B lines are present in diffuse and bilateral chest areas.^6,7^

Although there are well-defined protocols for LU execution and AIS definition, the examiner-dependent effect of LU can affect case management. This discrepancy arises from several factors, such as different times of exam performance, acute deterioration of the respiratory condition, technique applied, or even different delimitations of the assessed chest regions.^8^

The objective of this study is to evaluate the agreement between different examiners who received the same bedside LU training in their identification of the presence of B lines in to verify the reproducibility of the method.

## METHODS

This was a cross-sectional, observational, hospital-based study. Critical patients with some respiratory deterioration who were admitted to the adult intensive care unit (ICU) of *Hospital Felício Rocho* (HFR), located in Belo Horizonte, Minas Gerais, Brazil, were included in the study. The data were collected over a period of approximately 5 months (November 2016 to March 2017) after approval by the Research Ethics Committee of HFR. Prior to the exam, the patients participating in the study, or the responsible family member in the case of patients with compromised autonomy, signed an informed consent form.

Patients who were older than 18 years and had some ventilatory deterioration of clinical and/or surgical cause were eligible. Pregnant women or patients with trauma-related ventilatory deterioration were excluded from the study. After selection, the patients received two different LUs that were performed separately, each by a different intensive care physician. To perform the LU, the ventilatory deterioration should have occurred within a time interval shorter than 12 hours after the first exam was performed. This time interval was considered to enable the performance of the exam by different physicians. However, the time interval between each LU was 3 hours at most to reduce a possible loss of reliability in execution related to the time that had elapsed between the exams.

The patient's clinical condition, the cause of ventilatory deterioration and the results of the LUs were not known by the examining physicians to minimize information bias. To this end, another professional who did not directly participate in the study but who was knowledgeable of the inclusion criteria was responsible for selecting the eligible patients.

Ventilatory deterioration was defined as the occurrence of tachypnea (respiratory rate greater than 20 breaths per minute) possibly accompanied by respiratory effort (use of accessory muscles, broken speech and fatigue) and/or desaturation (drop below 90% in oxygen pulse saturation or need for increased basal oxygen flow to maintain saturation above 90%) when breathing spontaneously. In patients on mechanical ventilation, ventilatory deterioration was present when there was an increase/change in the device settings for better ventilatory dynamics and/or when there was a ventilator weaning failure from a respiratory apparatus-related cause, excluding ventilatory deterioration related to the absence of central ventilatory drive, isolated muscle weakness or to circuit/ventilator-related defects. Both changes had to be sustained and outside of the typical pattern for the patient in order to be included, i.e., occasional changes and/or changes related only to mishaps in patient monitoring were not considered.

Each LU was performed at the bedside, in real time and independently by the examiners: each result obtained with the LU was recorded in an individual form by each examining physician so that one examiner had no knowledge of the results of the others.

Three intensive care physicians (A, B and C) were selected because they had received similar training based on a 20-hour theoretical and practical course of the Intensive Care Society of Minas Gerais (*Sociedade Mineira de Terapia Intensiva* - SOMITI), called Ultrasonography for Intensive Care Physicians, which addresses ultrasonography as applied to critical patients. This course offers a practical discussion on pulmonary assessment for the differential diagnosis of dyspnea in critical patients, techniques for ultrasound-guided insertion of vascular access devices and diagnosis of deep vein thrombosis that is linked to a direct evaluation of cardiac function, in addition to abdominal assessment focusing on hemoperitoneum. The participating professionals had an average length of experience of five years in intensive care and routinely performed this diagnostic modality in their daily practice, with at least 2 years of experience with this tool. Since it was impossible for the three physicians to perform the examination on the same patient at the same time, three subgroups of two physicians each were created: AB, AC and BC.

To execute the LU, the patient was positioned in dorsal decubitus, with the head inclined approximately 30º and the chest exposed. A curved transducer was used at a frequency of up to 6MHz and positioned longitudinally and perpendicular to the chest. The gain (wave brightness amplification) and depth were modified according to the examiner's needs. The only chest zones that were evaluated were the anterior and lateral, which were defined as the right and left anterior (1) zones (Z1R and Z1L, respectively) and were delimited by the clavicle, sternum and the horizontal line perpendicular to the xiphoid process and anterior axillary line. The right and left lateral (2) zones (Z2R and Z2L, respectively) covered the lateral area between the anterior and posterior axillary lines and had as their lower limit the same horizontal line corresponding to the height of the xiphoid process.

Each zone was evaluated for the presence or absence of B lines. A "positive" zone was considered when three or more B lines were visualized, and a "negative" zone had a smaller number of or absent B lines. When there were doubts about the visualization of B lines, the term "uncertain" was accepted. All of the results that were found by each examiner were recorded in their own form and were not viewed by the other examiners (Appendix A). The definition of B line that was used was the same as that used by the BLUE protocol^7^ - vertical artifacts that erase A lines, are hyperechoic, arise from the superior pleural line and move with lung sliding.

The duration of the LU was measured for later comparison between the examiners. In addition, for comparison purposes, the average time required to perform an urgent chest radiography in the ICU patients was surveyed.

### Statistical analysis

The sample size was calculated from categorical variables. The estimated expected proportion of the presence of B lines in critical patients with ventilatory deterioration was equal to 0.20, while the range of the desired confidence interval was equal to 0.1 (0.05 above or 0.05 below) at a confidence interval equal to 95% (95%CI) and sample error of 8%. These variables resulted in a minimum sample size of 65 patients.^8^

The results that were found by the subgroups (AB, AC and BC) were compared using two-tailed statistical tests at a significance level of 5%. Variables were evaluated using the Kappa method, in which their interpretation is attributed a Kappa value that can vary from zero to 1. An interexaminer agreement level equal to zero represents no agreement, and a level equal to 1 represents perfect agreement. The intermediate values range from low (0 - 0.20), reasonable (0.21 - 0.39), moderate (0.40 - 0.59) to substantial (0.60 - 0.79).

## RESULTS

**Figure 1:**
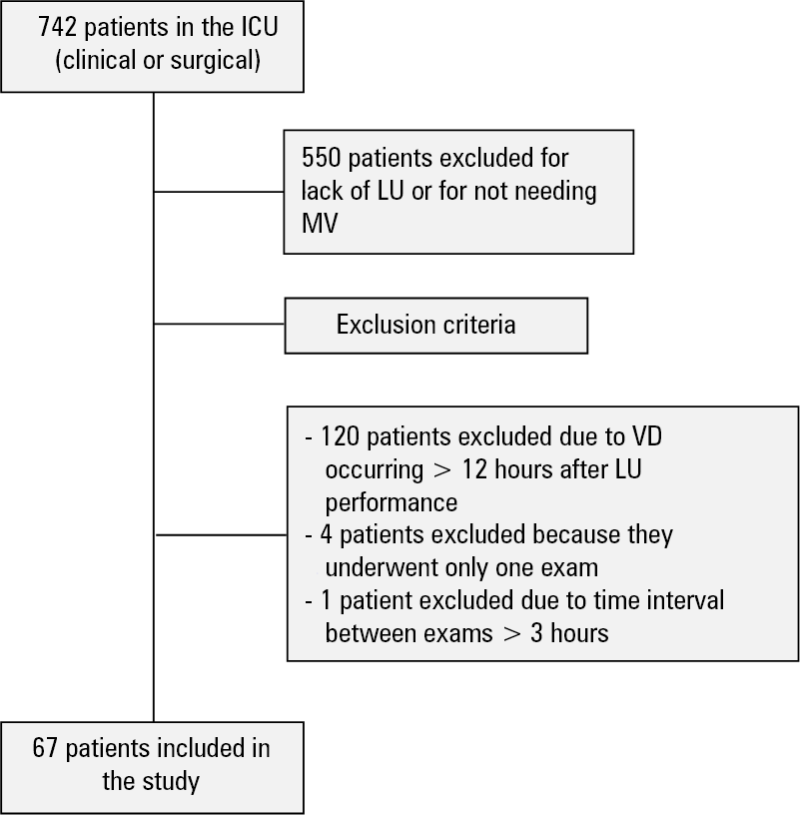
Flowchart of the exclusion criteria. ICU - intensive care unit; VD - ventilatory deterioration; MV - mechanical ventilation; LU - lung ultrasound.

Over the data collection period, 742 patients were admitted to this ICU. The population eligible for participation in the study was 192 adults. Of these, 120 were excluded because their ventilatory deterioration occurred in a time interval longer than 12 hours after the LU. Of the remaining 72 patients, four were excluded because they underwent only one LU, precluding the calculation of agreement, and one was excluded because the interval between each LU was greater than 3 hours, thus leaving 67 patients who were included in the study (Figure 1).

There was a predominance of males (58%) in the patient sample that was analyzed; the mean age was approximately 68 years for the males and was slightly higher for the females (69 years).

More than 30 comorbidities were found; the main ones related to a possible LU abnormality were systemic arterial hypertension, which affected 34 patients (50.7%); congestive heart failure in 19 patients (28.4%); and chronic obstructive pulmonary disease in six patients (8.9%). With regards to the type of ventilation of the included patients at the time of LU, 45 (67.1%) were breathing spontaneously and 22 (32.9%) were on mechanical ventilation (Table 1).

**Table 1:** Major comorbidities of the evaluated patients

Comorbidities	%
Hypertension	50.7
Malignant neoplasm	29.9
*Diabetes mellitus*	28.4
Heart failure (EF > 40%)	16.4
Heart failure (EF < 40%)	11.9
Chronic kidney disease	22.4
Chronic obstructive pulmonary disease	8.9

EF - ejection fraction.

The data collected in each subgroup used the same inclusion and exclusion criteria. The AB, BC and AC subgroups performed 23, 20 and 24 LUs, respectively, for a total of 67 LUs. The Kappa coefficient between each subgroup (AB, AC and BC) for each zone (Z1R, Z2R, Z1L and Z2L) was calculated with its 95%CI.

The highest levels of agreement occurred in the anterior zones, both between the AC and BC subgroups, with a Kappa coefficient of agreement greater than 0.6. In examiners A and B, the highest agreement occurred in zone Z2L, with a Kappa of 0.611, followed by zone Z1R, with a Kappa of 0.495, and zone Z2R, with a Kappa of 0.469, indicating moderate agreement (0.40 to 0.59).^9^ For zone Z1L, the agreement was only reasonable (0.20 to 0.39),^9^ as it had a Kappa of 0.321 and was statistically nonsignificant (p > 0.05)

In the AC subgroup, there was significant agreement for all zones (p < 0.05). The highest levels of agreement between examiners (A and C) occurred in zone Z1L, with a Kappa of 0.657, and zone Z1R, with a Kappa of 0.647, indicating substantial agreements (0.60 to 0.79);^9^ zone Z2R, with a Kappa of 0.525, and zone Z2L, with a Kappa of 0.438, demonstrated moderate agreement (0.40 to 0.59) in this subgroup.^9^

In the BC subgroup, there was significant agreement for practically all zones (p < 0.05) except for Z1R. The highest levels of agreement between examiners (B and C) occurred in zones Z1L, with a Kappa of 0.792, indicating substantial agreement (0.60 to 0.79)^9^ and zones Z2R, with a Kappa of 0.412, and Z1R, with a Kappa of 0.406, indicating moderate agreement (0.40 to 0.59). For zone Z2L, which had a Kappa of 0.365, the agreement was reasonable (0.20 to 0.39),^9^ as shown in table 2.

**Table 2:** Kappa agreement index between the subgroups

Subgroup	Areas	Kappa (95% CI)	Degree of agreement	p value
AB	Z1R	0,495	Moderate	0.011
	Z1L	0,321	Reasonable	0.059
	Z2R	0,469	Moderate	0.006
	Z2L	0,611	Substantial	0.000
	Average agreement between zones: 0.474			
AC	Z1R	0,647	Substantial	0.001
	Z1L	0,657	Substantial	0.001
	Z2R	0,525	Moderate	0.004
	Z2L	0,438	Moderate	0.002
	Average agreement between zones: 0.567			
BC	Z1R	0,406	Moderate	0.064
	Z1L	0,792	Substantial	0.000
	Z2R	0,412	Moderate	0.047
	Z2L	0,365	Reasonable	0.027
	Average agreement between zones: 0.494			

95% CI - 95% confidence interval

The mean duration of LU among the three subgroups was 2.5 minutes, ranging from 40 seconds to 3 minutes. The time interval between the two LUs was less than one hour in 57 patients (85%). In the remaining 10 patients, this interval ranged from 2 to 3 hours, and in half (five patients), there was full agreement among the examiners.

The mean duration of the urgent chest radiography, including the availability of the images in a computerized system, was 25 minutes, according to the information that was obtained in the radiology department of HFR.

## DISCUSSION

The analysis showed that the mean agreement of all three subgroups was moderate (Kappa 0.40 - 0.59), suggesting the good reproducibility of the method, considering the satisfactory sample (sampling error fixed at 8%) and suggesting that it can be performed in real time.^8,10,11^ The subgroup analysis strengthens the reliability of the LU because in each subgroup, most zones had a moderate to substantial Kappa value, ranging from 0.41 to 0.79 (p < 0.05).^7^ The left zones (Z1L and Z2L) showed low agreement between some subgroups, probably because this region coincides with the cardiac area, generating image overlap and hindering evaluation.^11-13^

As proposed by some studies, the presence of three or more B lines in the diffuse and bilateral thoracic chest areas suggests AIS, which could result from pulmonary fibrosis, cardiogenic pulmonary edema or acute respiratory distress syndrome.^6,7,12-15^ Since the focus of this study is to assess the reliability of an examiner-dependent method in a real clinical setting and not its diagnostic accuracy, it was decided to qualitatively categorize the presence of B lines (positive, negative or uncertain) because this allows for the presence of pathologies to be inferred.^8,9,16^

The technical skills and experience of each examining physician were considered satisfactory because they had taken the same course and had a minimum of 2 years of experience with this diagnostic modality.^17^ See et al. showed that respiratory therapists acquired competence in performing LU after a few hours of theoretical training, performing the exam adequately and without the need for supervision after approximately ten guided LUs.^18^

In addition, 85% of patients had a time interval less than an hour between each LU, i.e., most of the two exams performed for the same patient occurred within an interval of less than 60 minutes, reducing the possibility of unreliability due to actions that could be taken in this time interval or due to disease progression. In addition, half of the remaining evaluated patients had an interval of 2 - 3 hours between LUs; even so, there was total agreement between the LUs in the evaluation, suggesting the reproducibility of the method.

The duration of LU was brief because its aim was to provide an objective and dichotomous answer for fast decision-making. This time interval was similar to that found in the BLUE protocol^6^ but was three times lower than that of a similar study that was also performed in real time.^15,19^ The probable reason for this variation is the greater number of chest zones that were analyzed in the latter study, thus requiring more time from the examiner.

Comparing the mean time required for an urgent chest radiograph with the speed at which an LU can be performed in settings where the device is available full time, there are clear benefits from choosing the LU as the first evaluation method of respiratory changes in critical patients, considering, above all, its innocuity to patients. Regarding the reduction in harm and increased safety for patients, a study conducted in Italy found that the use of LU was associated with a 26% reduction in the total number of chest radiographs and a 47% reduction in the total number of computerized tomographies.^15^

Our study has some limitations. The ideal study would have the three examiners perform an LU on the same patient rather than subdividing them into groups. However, the logistical difficulty of gathering the three professionals at the time of ventilatory deterioration justifies this separation.

Another point of negative impact in this study was the item "uncertain", which was intended for the visualization of B lines since it was also included in the analysis of agreement. It was not established whether the term "uncertain" could be related to changes in the lung parenchyma image or to the difficulties generated by the identification of underlying structures such as the heart, pleural effusion and hepatomegaly. This observation may explain the lower Kappa value in the AB subgroup, since the "uncertain" rate was the highest detected, with this result in at least one lung zone of each patient.

The analysis of the chest zones in quadrants, and not in a punctiform manner, may also have contributed to the disagreement in diagnoses in some cases, despite the standardized patient position for LU. Evaluating larger areas may have led to difficulties in the interpretation of some patients due to anatomical changes as well as to the severity of the disease. In a recent narrative review, disagreements among examiners in the diagnostic evaluation of chest tomographies in patients with suspected community-acquired pneumonia occurred in more than 40% of cases.^20^ Finally, other points to be reviewed would be the recording and archiving of LU examinations for their later analysis or by other examiners, evaluating whether the interpretation would have been made and/or maintained using the same image.

## CONCLUSION

Our study showed that the average agreement among the three subgroups was identified as moderate by the Kappa value, indicating a good consistency of the lung ultrasonography results when repeated by different examiners. The inclusion criteria and performance of the exams in a similar manner to the actual clinical scenario suggest the good reproducibility of this diagnostic method.
